# Effects of neoadjuvant chemotherapy on the contralateral non-tumor-bearing breast assessed by diffuse optical tomography

**DOI:** 10.1186/s13058-021-01396-w

**Published:** 2021-01-31

**Authors:** Mirella L. Altoe, Kevin Kalinsky, Alessandro Marone, Hyun K. Kim, Hua Guo, Hanina Hibshoosh, Mariella Tejada, Katherine D. Crew, Melissa K. Accordino, Meghna S. Trivedi, Dawn L. Hershman, Andreas H. Hielscher

**Affiliations:** 1 Departments of Biomedical Engineering, New York University Tandon School of Engineering, Brooklyn, NY 11201 USA; 2 Department of Medicine, Division of Hematology/Oncology, Columbia University Irving Medical Center, New York, NY 10032, USA; 3 Department of Pathology and Cell Biology, Columbia University Irving Medical Center, New York, NY 10032 USA; 4 Department of Epidemiology, Columbia University Irving Medical Center, New York, NY 10032 USA

**Keywords:** Diffuse optical tomography, Breast cancer, Contralateral breast, Menopausal status, NAC, pCR

## Abstract

**Background:**

The purpose of this study is to evaluate whether the changes in optically derived parameters acquired with a diffuse optical tomography breast imager system (DOTBIS) in the contralateral non-tumor-bearing breast in patients administered neoadjuvant chemotherapy (NAC) for breast cancer are associated with pathologic complete response (pCR).

**Methods:**

In this retrospective evaluation of 105 patients with stage II–III breast cancer, oxy-hemoglobin (*ctO*_2_*Hb*) from the contralateral non-tumor-bearing breast was collected and analyzed at different time points during NAC. The earliest monitoring imaging time point was after 2–3 weeks receiving taxane. Longitudinal data were analyzed using linear mixed-effects modeling to evaluate the contralateral breast *ctO*_2_*Hb* changes across chemotherapy when corrected for pCR status, age, and BMI.

**Results:**

Patients who achieved pCR to NAC had an overall decrease of 3.88 μM for *ctO*_2_*Hb* (95% CI, 1.39 to 6.37 μM), *p* = .004, after 2–3 weeks. On the other hand, non-pCR subjects had a non-significant mean reduction of 0.14 μM (95% CI, − 1.30 to 1.58 μM), *p* > .05. Mixed-effect model results indicated a statistically significant negative relationship of *ctO*_2_*Hb* levels with BMI and age.

**Conclusions:**

This study demonstrates that the contralateral normal breast tissue assessed by DOTBIS is modifiable after NAC, with changes associated with pCR after only 2–3 weeks of chemotherapy.

## Background

Neoadjuvant chemotherapy (NAC) has become a well-established therapy in treating patients with locally advanced breast cancer and consists of 3–6 months of systemic, preoperative treatment. Chemotherapy can modify the density and composition of normal breast tissue. Standardized response evaluation after NAC can be assessed by residual cancer burden (RCB) index, which is quantified based on primary tumor area, percentage of the tumor area that is invasive cancer, and extent of lymph node involvement. A score of 0 (RCB0) equates to a pathological complete response (pCR) to NAC, and it is defined as the complete disappearance of the tumor in the breast and lymph node (ypT0/is ypN0 Mx) at the time of surgery. Non-pCR tumors can be categorized into one of the following three classes: RCB-I (minimal burden), RCB-II (moderate burden), and RCB-III (extensive burden). RCB after NAC is associated with improved clinical outcomes, such as disease-free survival, and can predict disease recurrence across different breast cancer subtypes [1–3].

Relatively inexpensive, simple to operate, and well-tolerated by patients, diffuse optical tomography (DOT) has shown promising applications in breast cancer detection and monitoring NAC [4–8]. However, the measurement of optically derived parameters in the affected breast is strongly dependent on the tumor location, and DOT suffers from low spatial resolution due to its nonlinear, ill-conditioned, and ill-posed inverse problem [9]. Using a priori information provided by an additional high-resolution anatomical imaging modality, such as magnetic resonance or X-ray, could improve spatial resolution but also nullify the low-cost benefit associated with DOT techniques [10]. Therefore, DOT quantitative measure from the contralateral breast could offer a non-tumor dependent measurement to monitor NAC induced changes in the breast tissue and assess treatment outcome. Evaluation of optically derived parameters variation in the contralateral non-tumor-bearing breast following NAC has been previously analyzed [11]. Still, no extensive analyses have been performed to evaluate whether these changes are associated with tumor response in the affected breast. A study published in 2005 [12] reported that chemotherapy could decrease contralateral breast cancer risk in younger women [13]. However, assessment of chemotherapy-related benefits for developing a newly detected breast cancer is still a challenge due to the absence of accurate quantitative metrics.

Over the last decade, our group has developed a diffuse optical tomography breast imaging system (DOTBIS) for a full 3-dimensional tomographic assessment of the entire breast volume and simultaneous bilateral breast imaging. DOTBIS data acquisition is performed without contrast agents or compression and can measure tissue concentration of oxy-hemoglobin (*ctO*_2_*Hb*), deoxy-hemoglobin (*ctHHb*), and water. Our previous studies have described the utility of DOTBIS in monitoring tumor response to NAC [7, 14] and tumor detection [15]. As our study was performed bilaterally on breast cancer patients, DOTBIS quantitative measurements from the contralateral non-tumor-bearing breast were acquired and reported in this paper.

The purpose of this study is to investigate the change of quantitative measurements of functional tissue components, such as hemoglobin concentration and water percentage, measured with DOTBIS in the contralateral non-tumor-bearing breast of patients receiving NAC. We hypothesize that hemoglobin and water values in the unaffected breast, directly related to tissue metabolism and vascular characteristics, are associated with pCR to NAC.

## Methods

Between 2011 and 2019, 105 patients diagnosed with stage II–III breast cancer and scheduled to undergo NAC were consented to participate in a DOTBIS longitudinal research. Menopausal status, body mass index (BMI), and age were retrieved from the patient’s electronic medical record. pCR in the breast was defined as the absence of invasive carcinoma (ypT0/is ypN0 Mx) at the time of surgery. Residual Center Burden (RCB) was assessed per established guidelines by Symmans et al. [16]. This study was approved by the institutional review board at Columbia University Irving Medical Center (CUIMC).

### DOTBIS imaging acquisition

**Fig. 1:**
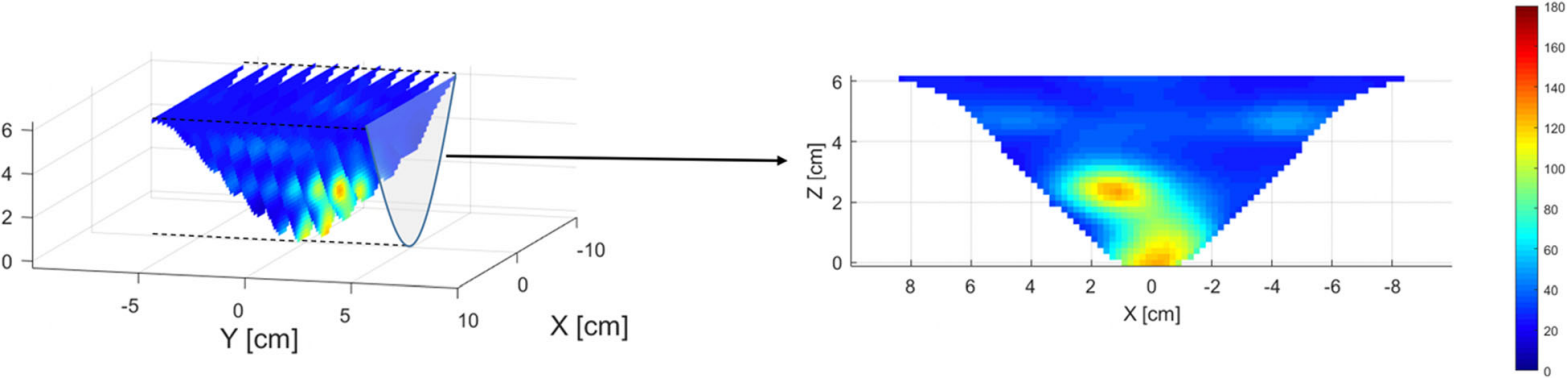
Visual scheme of how maximum intensity projection (MIP) images are generated from the DOTBIS acquired *ctTHb* 3D map (μM). MIP is obtained by finding the maximum intensity for each voxel along the *y*-axis direction. The maximum voxel values are registered into a single view plane for visualization purposes only

Measurements were performed by a continuous wave dynamic imaging system (i.e.*,* DOTBIS) that provides full three-dimensional tomographic images of both breasts simultaneously without requiring the use of contrast agents or compression. A more detailed description of the system can be found in previous reports [15, 17]. Total measurement time varied between 6 and 8 min in addition to 5 min required to set up the machine. Near-infrared light transmission was carried out using four laser diodes operating at 765, 808, 827, and 905 nm. The patient interface consists of two sets of four rings that use up to 32 sources and 64 detectors, depending on the patient’s breast cup size, for optical data collection. The spatial distribution of chromophore concentration of *ctO*_2_*Hb*, *ctHHb*, and water was obtained by a transport-theory-based PDE-constrained multispectral model [18], which uses the diffusion approximation of the equation of radiative transfer to describe the light propagation in such scattering-dominated media as the breast tissue. Additional features such as *ctTHb* and *StO*_2_% were defined respectively as Eq. (1) and Eq. (2). For visualization, maximum intensities projections (MIP) [19, 20] were obtained from the reconstructed 3D chromophore concentration maps. MIP projects the acquired data volume into a view plane, which can be obtained by finding the voxels with maximum intensity along a chosen axis, Fig. 1.

1 $$ ctTHb= ct{O}_2 Hb+ ctHHb $$

2 $$ {StO}_2\%=\frac{ct{O}_2 Hb}{ ct THb}\times 100\% $$

### NAC imaging protocol

**Fig. 2:**
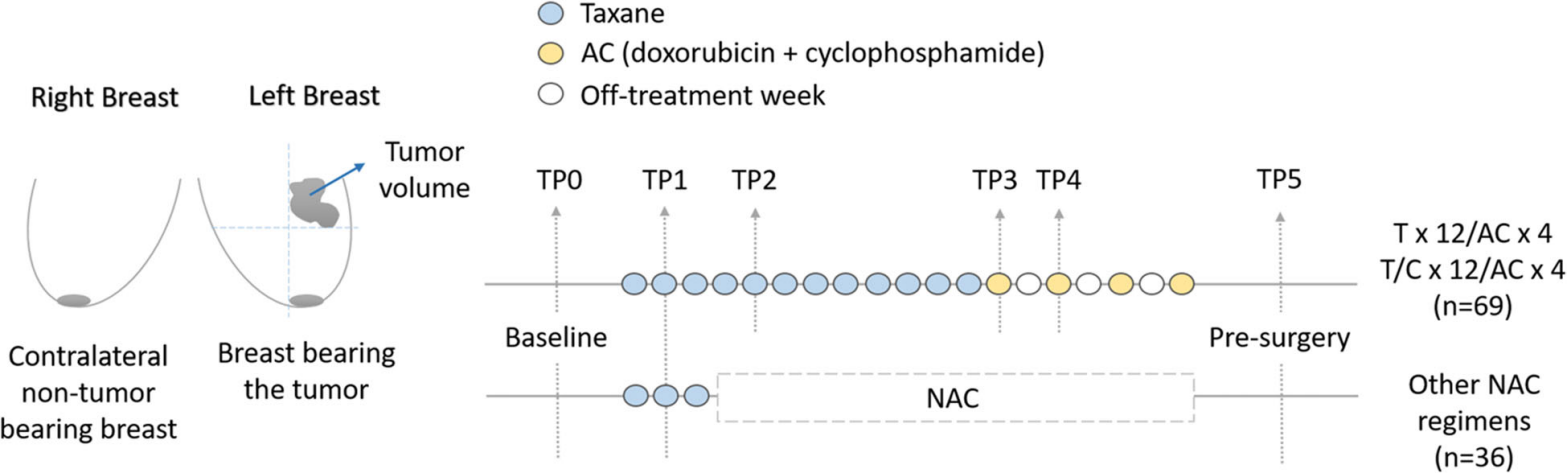
DOTBIS bilateral measurements were acquired at six or three different time points depending on the NAC regimen. For patients under T × 12/AC × 4 and T/C × 12/AC × 4 therapies (*n* = 69), contralateral non-tumor-bearing breast and the breast bearing the tumor were imaged at baseline (TP0), 2 weeks after the first taxane infusion (TP1), after four infusions of taxane (TP2), at the end of the taxane regimen and before starting AC cycle (TP3), after two AC infusions (TP4), and at the end of NAC and before surgery (TP5). For other taxane-based NAC regimens (*n* = 36), both breasts were imaged only three times: TP0, 2–3 weeks after the first taxane infusion (TP1), and TP5

Sixty-nine patients received 12 cycles of weekly paclitaxel (with or without the addition of carboplatin) followed by four cycles of doxorubicin and cyclophosphamide given every 2 weeks with growth factor support (T × 12/AC × 4 or T/C × 12/AC × 4), and DOTBIS bilateral measurement was acquired at six different time points: before starting NAC regimen (TP0), after 2 weeks of taxane infusions (TP1), after 4 weeks of taxane (TP2), at the end of the taxane regimen and before starting AC cycles (TP3), after two cycles of AC (TP4), and at the end of NAC and before surgery (TP5). Thirty-six patients received other taxane-based NAC regimens without an anthracycline, and bilateral imaging was performed only three times (baseline (TP0), 2–3 weeks after the first taxane infusion (TP1), and at the end of NAC and before surgery (TP5)), Fig. 2.

### Statistical analysis

An independent-samples *t* test was run to determine if there were differences in baseline DOTBIS parameters between pre-and postmenopausal subjects and pCR status (pCR and non-pCR patients). Feature importance was calculated by using Random Forest [21] model-specific metrics from the R package “caret” [22]. The Random Forest algorithm has a built-in feature selection, mean decrease Gini (Gini importance) [23], which can be used as a score that provides a relative ranking of feature relevance. A paired-samples *t* test was used to determine whether there was a statistically significant mean difference in *ctO*_2_*Hb* levels measured at baseline and TP1 between pCR and non-pCR and between both menopausal groups. To account for repeated measures, a linear mixed-effect model was designed to evaluate if there is a difference in the average longitudinal evolution of *ctO*_2_*Hb* when we correct for pCR status (or age and BMI). Significance was assumed at a confidential interval of 95% (α = 0.05).

## Result

### Patient summary

From a total of 105 subjects, 18 were excluded from the final analysis data set. The main reasons for exclusion were as follows: patient withdrew from the study (*n* = 4), baseline DOTBIS measurement not collected due to instrumentation failure (*n* = 8), patient receiving non-FDA approved drug regimen (*n* = 1), bilateral breast cancer (*n* = 1), and machine operator error (*n* = 4). Not all the patients had their DOTBIS reconstruction available for all of their imaging time points due to patient declining imaging at that particular DOTBIS time point or machine malfunction. Overall, 81 (93.1%) patients had DOTBIS images successfully reconstructed at baseline (TP0), 69 (79.3%) patients at 2–3 weeks from taxane initiation (TP1), and 52 (59.8%) at the pre-surgical time point (TP5). Missing TP5 data points were due to patient declined to be imaged (18/35), machine operator did not collect/save the data correctly (5/35), and data not collected due to machine under repair (12/35). Out of the 58 patients who were administered the T × 12/AC × 4 (*n* = 54) regimen or T/C × 12/AC × 4 (*n* = 4), 46 (79%) had their data acquired and reconstructed at TP2, 43 (74%) at TP3, and 40 (69%) at TP4. Non-anthracycline regimens accounted for the remaining 29 patients. Patient and clinical characteristics are summarized in Table 1.

**Table 1:** Patient and clinical characteristics for all the 87 patients considered for analysis

Patient and clinical characteristics	Number of patients	Age (mean ± SD)	BMI (mean ± SD)
Chemotherapy and targeted therapies			
T × 12/AC × 4	54	48.48 ± 11.52	29.89 ± 7.18
TCHP × 6	21	53.80 ± 10.04	29.307 ± 9.49
TC × 6	6	59.67 ± 12.24	30.54 ± 3.24
T/C × 12/AC × 4	4	39.25 ± 8.65	30.05 ± 2.29
TC × 4	1	30	18.11
THP × 12/AC × 4	1	40	32.23
Menopausal status			
Premenopausal	52	41.65 ± 6.90	29.48 ± 7.91
Postmenopausal	34	61.63 ± 6.00	30.10 ± 6.82
Uncertain	1	56	27.16
**Tumor characteristics**	**Number of tumors**		
Treatment response classification			
pCR			
RCB 0	36		
Non-pCR			
RCB I	10		
RCB II	27		
RCB III	11		
RCB score not available	3		

Abbreviations and acronyms: T × 12/AC × 4, 12 cycles of weekly paclitaxel followed by 4 cycles of doxorubicin and cyclophosphamide given every 2 weeks with growth factor support; T/C × 12/AC × 4, 12 cycles of weekly paclitaxel followed by 4 cycles of doxorubicin and cyclophosphamide given every 2 weeks with growth-factor support with an addition of carboplatin to paclitaxel; TC × 6, six cycles Cytoxan plus Taxotere; TC × 4, four cycles Cytoxan plus Taxotere; TCHP, six cycles of docetaxel, carboplatin, and trastuzumab plus pertuzumab; THP/AC, paclitaxel, trastuzumab, and pertuzumab followed by doxorubicin and cyclophosphamide; pCR, pathologic complete response; RCB, residual cancer burden; BMI, body mass index; SD, standard deviation

### Baseline DOTBIS-measured features

Contralateral *ctTHb*, *ctO*_2_*Hb*, *ctHHb*, water, and *StO*_2_ values were available for all the 87 patients considered for analysis at different time points across NAC. At the baseline level, there was no statistical difference in any of the DOTBIS-measured features between pCR and non-pCR patients (*p* > 0.05). Fifty-two patients (59.8%) were premenopausal women, 34 (39.1%) were postmenopausal, and for one patient (1.1%), her menopausal status was uncertain. An independent samples *t* test was run to determine if there were differences in baseline DOTBIS parameters between pre-and postmenopausal subjects. Pre-menopausal women had higher mean *ctTHb* (33.30 ± 12.22 μM) than post-menopausal women (24.92 ± 7.78 μM), a statistically significant difference of 8.39 μM (95% CI, 3.96 to 12.81), t(79) = 3.772, *p* < .0005. *ctO*_2_*Hb* levels were also higher for premenopausal women (19.36 ± 8.51 μM) than postmenopausal (13.24 ± 4.89 μM). The difference in means for water was also statistically significant between pre- (48.37 ± 9.33%) and postmenopausal (40.48 ± 12.14%) patients, *p* = .001.

### Contralateral breast DOTBIS feature selection

To avoid severe multicollinearity or overfitting, variable importance with random forest was performed to find the most relevant pCR status classification feature. Looking at the changes of all the DOTBIS-measured features after 2–3 weeks under NAC, *ctO*_2_*Hb* was selected as the best predictor characterized by the highest mean decrease impurity (Gini importance). For this feature, lower values are associated with poor response to NAC (non-pCR).

### *ctO*_2_*Hb* changes in the contralateral breast after 2–3 weeks under NAC and pCR status

**Fig. 3:**
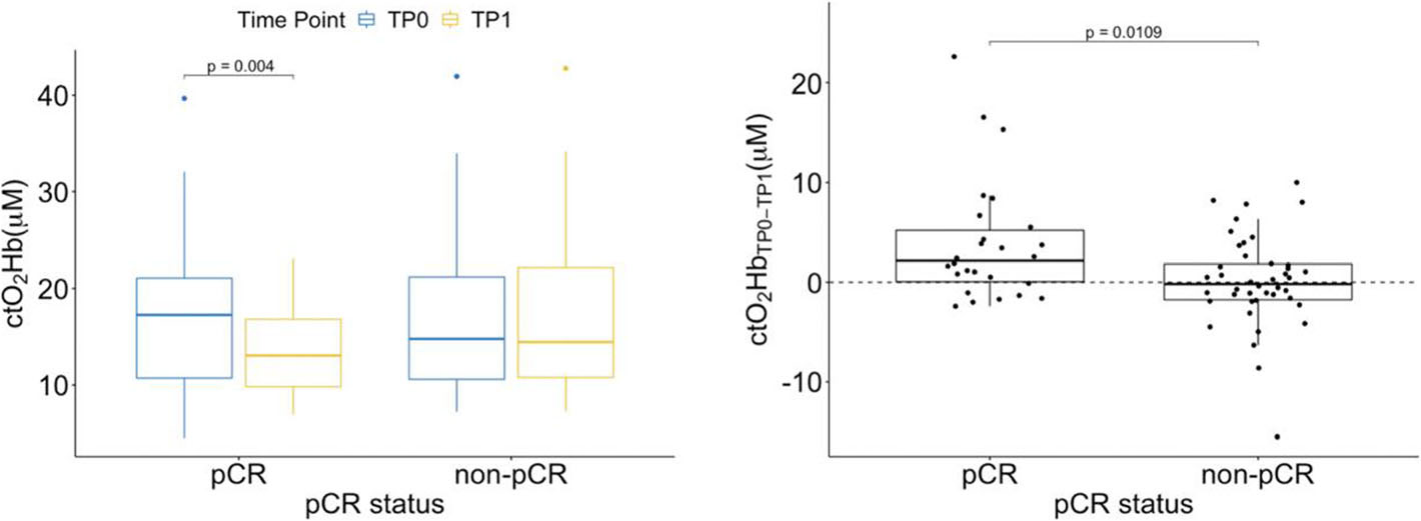
Left: *ctO*_2_*Hb* changes after 2–3 weeks of chemotherapy showing a significant decrease for pCR patients but a non-significant change for the non-pCR group. Right: Box plot of the difference between *ctO*_2_*Hb* levels measured at baseline and after 2–3 weeks under Taxane regimen (TP1) for pCR and non-pCR patients showing a significant difference between both groups. *ctO*_2_*Hb* reduction levels at TP1 were larger for pCR patients

Thirty-seven subjects (42.5%) achieved pCR and 50 (57.5%) were classified as non-pCR. A paired *t* test was used to determine whether there was a statistically significant mean difference between baseline *ctO*_2_*Hb* and after 2–3 weeks under NAC (TP1). Overall, NAC elicited a mean decrease of 1.57 μM for *ctO*_2_*Hb* levels (95% CI, 0.28 to 3.32 μM), t(67) = 2.43, *p* = .022, in the contralateral non-tumor-bearing breast. Looking at the pCR group, NAC elicited an overall decrease of 3.88 μM for *ctO*_2_*Hb* (95% CI, 1.39 to 6.37 μM), *p* = .004. On the other hand, the non-pCR subjects had a non-significant mean reduction of 0.14 μM for *ctO*_2_*Hb* (95% CI, − 1.30 to 1.58 μM), *p* = .845, Fig. 3.

### *ctO*_2_*Hb* longitudinal analysis in the contralateral breast during NAC: pCR status, age, and BMI as model predictors

**Fig. 4:**
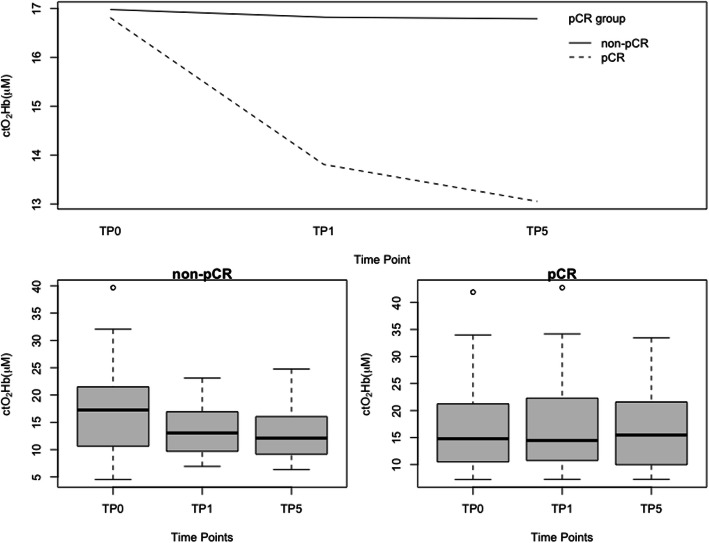
Top: Plot illustrates the interaction between the mean response for two-way combination of factors: time and pCR status over three-time points. Bottom: Box plots and 95% confidence intervals for *ctO*_2_*Hb* separated by tumor response to NAC: pCR (Left) non-pCR (Right) groups

**Fig. 5:**
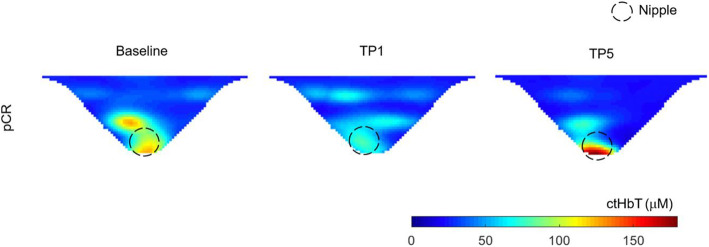
Corresponding hemoglobin levels map for the contralateral unaffected breast of 47-year-old women who achieved pCR after TCHP therapy for HER2+ breast cancer. MIP images (axial orientation) were acquired at baseline, after 2–3 weeks after NAC initiation (TP1), and at the pre-surgical time point (TP5)

**Fig. 6:**
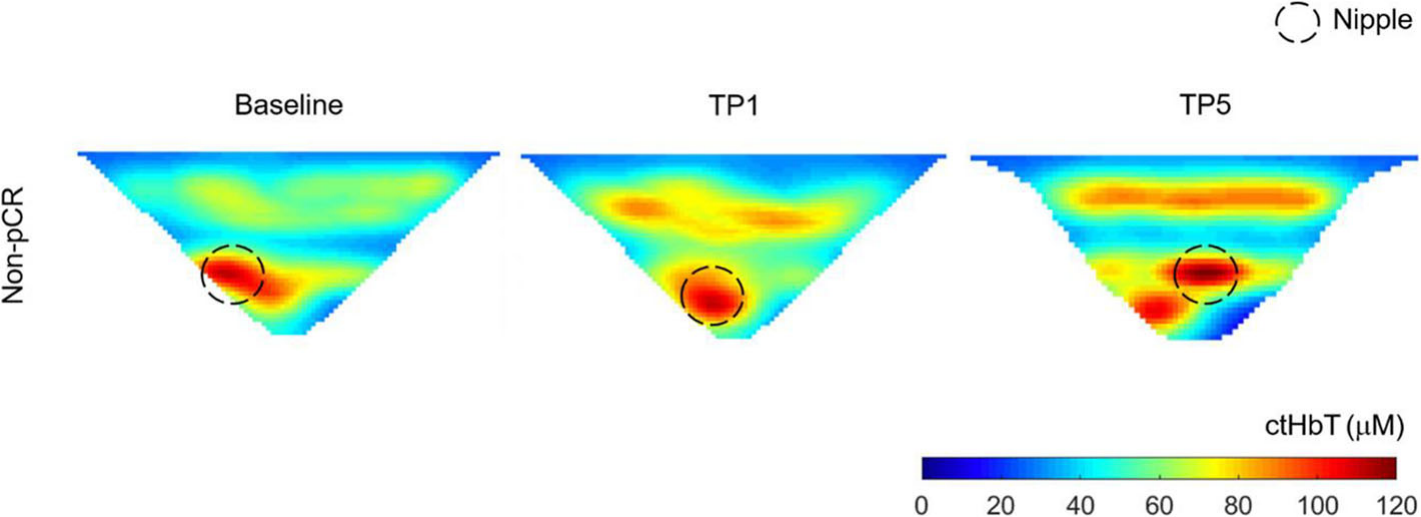
Corresponding hemoglobin levels map for the contralateral unaffected breast of 46-year-old women classified as RCB III (non-pCR) after Tx12/ACx4 therapy for HER2- breast cancer. MIP images (axial orientation) were acquired at baseline, after 2–3 weeks of NAC (TP1), and at the pre-surgical time point (TP5)

For the longitudinal multilevel mixed-effects analysis, *ctO*_2_*Hb* from all patients (n = 87) was analyzed at three-time imaging points, TP0, TP1, and TP5, which are the imaging time points in common to all the patients regardless of their chemotherapy regimen. We fit a linear mixed-effects model with *ctO*_2_*Hb* as the outcome variable, with fixed effects of pCR status, BMI and age, and time as a random effect (and fixed effect) and random slope across individuals. We were also particularly interested in the group-occasion interaction between time and pCR status, as this reflects a potential difference in time evolution between the pCR status groups. There was a significant interaction between *ctO*_2_*Hb* over time and pCR status. pCR patients had a significant reduction in *ctO*_2_*Hb* levels at TP1 (3.53 μM, *p* = .0053) than non-pCR, but not statistically significant at TP5 (2.59 μM, *p* = .131). This trend can also be observed in the iteration plot of *ctO*_2_*Hb* levels over the different time points for both pCR and non-pCR groups, Fig. 4. Significant main effects of age (*p* = .0015) and BMI (*p* = .0011) were also found, Table 2, characterized by a negative relationship, older and overweighted patients had lower *ctO*_2_*Hb* levels. Figure 5 (pCR) and Fig. 6 (non-pCR) illustrate the hemoglobin map for two premenopausal patients imaged at three different time points during NAC regimen.

**Table 2:** Summary for the longitudinal model analysis: estimated model fixed effects

Effect	Estimate	Std. error	t	***p*** value
Intercept	33.41	3.65	9.15	< 0.0005
pCR status	0.76	1.61	0.47	0.64
Time TP1	− 3.60	0.97	− 3.72	0.0003
Time TP5	− 3.73	1.32	− 2.82	0.0056
BMI	− 0.29	0.008	− 3.37	0.0011
Age	− 0.17	0.052	− 3.39	0.0015
TP1: pCR status	3.53	1.24	2.84	0.0053
TP5: pCR status	2.59	1.70	1.52	0.1306

Abbreviations and acronyms: pCR, pathologic complete response; TP1, time point after 2–3 weeks under chemotherapy; TP5, time point after ending chemotherapy

## Discussion

We investigated whether the changes of DOTBIS features in the contralateral healthy breast are associated with therapy outcome in patients with stage II–III breast cancer receiving NAC. We observed that hemoglobin level is modifiable throughout the course of NAC. The magnitude of change is associated with pCR after only 2–3 weeks with the initiation of a taxane-based regimen. Patients who achieved a pCR to NAC had a more significant reduction in DOTBIS-measured features than the non-pCR group.

Previous studies have demonstrated statistically significant changes in optically derived parameters in breast cancer patients undergoing NAC and their correlation with tumor response [5, 14, 24–28]. However, most of these studies looked at the tissue composition of the tumor volume, and few of them only used the contralateral healthy breast data as a normalizing factor for the total tissue hemoglobin concentration within the tumor region. The measurement of optically derived parameters in the affected breast is strongly dependent on selecting the tumor location or region of interest (ROI). Yet, it is well known that breast optical imaging has limitations regarding spatial resolution. Therefore, the optically derived parameters collected from the contralateral unaffected breast can offer a more unbiased and non-ROI dependent measurement to follow up the course of changes in the breast tissue during NAC and associations with treatment outcome. The only two previous studies [11, 29], which evaluated optical imaging changes during NAC in the contralateral normal tissue, did not perform analyses regarding tumor response. By associating contralateral healthy breast data with tumor response, we could provide a potential marker for evaluating chemotherapy regimens’ efficacy early in the treatment course.

A similar association with pCR is also reported in the literature for background parenchymal enhancement (BPE) at breast magnetic resonance (MR) imaging in the contralateral normal breast. Several groups [30–33] have demonstrated an association between BPE and tumor response after receiving NAC in the last 4 years. Chen et al. found that BPE showed a significant decrease in those with pCR compared to non-pCR for pre/peri-menopausal patients after starting NAC [30]. Next, Preibsch et al. investigated the influence of chemotherapy on the degree of BPE and found correlations between tumor response and qualitative assessment of BPE following NAC [31]. You et al. also reported a positive association between overall BPE reduction at the early stage of NAC and tumor response, especially in patients with hormone receptor-negative breast cancers [32].

Given enough evidence that optical imaging is a powerful tool to study the breast tissue’s structural and vascular properties [34], *ctTHb* and *ctO*_2_*Hb* are thought to be correlated to the amount of blood flow in the fibroglandular tissue and represent breast vascular supply and perfusion. The significant large reduction of DOTBIS-measured features for the premenopausal group may be explained by the ovarian function suppression that affects estrogen secretion and leads to a secondary change in breast tissue composition and its vascular characteristics [35, 36]. A review published in 2005 [12] also concluded that younger women had much larger benefits from chemotherapy than older patients, potentially due to the ovarian ablation induced by chemotherapeutic agents. Fibroglandular tissue (FGT) and BPE-MRI were also reported as markedly influenced by menopausal status, significantly decreasing after menopause for most women included in the study [37]. On the other hand, substantial changes in DOTBIS-measured parameters for those with pCR regardless of their menopausal status could also be associated with the direct damage of the vascular morphology and density in normal tissue caused by chemotherapy, as investigated by Miller et al. [38]*.*

Besides, the mixed-effect model results indicated a statistically significant negative relationship between *ctO*_2_*Hb* and BMI. DOTBIS-measured *ctO*_2_*Hb* is thought to be correlated to breast density since it is related to the arterial blood supply carrying oxygen to meet the fibroglandular tissue metabolic demand [29]. In contrast, BMI is inversely related to percent density and acts as a co-founder of each other’s effects [39–42]. In other words, overweight is correlated to a greater amount of fat in the breast, reflecting a larger non-dense tissue area in the mammogram and, consequently, lower percent density classification [41].

A limitation of our study was we did not reliably capture the patient’s menstrual period during treatment and around the time of scans. Besides that, since the majority of the patients in our study did not have breast MR imaging before and after NAC treatment, we were not able to expand our analyses to correlations between MR based quantitative measurements and DOTBIS features. Finally, a more extensive study is also required to identify potential optical predictors of long-term outcomes in breast cancer patients who undergo NAC and understand chemotherapy’s effect on reducing contralateral cancer risk.

## Conclusion

In summary, this is the first study to confirm that optical derived metrics, measured by DOTBIS, in the contralateral non-tumor-bearing breast tissue, are modifiable with NAC treatment. *ctO*_2_*Hb* changes are proportional to the degree of pCR after 2–3 weeks of taxane-based regimen.

## Data Availability

The datasets used and analyzed during the current study are available from the corresponding author on reasonable request**.**
